# Hotter droughts alter resource allocation to chemical defenses in piñon pine

**DOI:** 10.1007/s00442-021-05058-8

**Published:** 2021-10-17

**Authors:** Amy M. Trowbridge, Henry D. Adams, Adam Collins, Lee Turin Dickman, Charlotte Grossiord, Megan Hofland, Shealyn Malone, David K. Weaver, Sanna Sevanto, Paul C. Stoy, Nate G. McDowell

**Affiliations:** 1 Department of Entomology, University of Wisconsin-Madison, Madison, WI 53706 USA; 2 Department of Land Resources and Environmental Sciences, Montana State University, Bozeman, MT 59715 USA; 3 School of the Environment, Washington State University, Pullman, WA 99164 USA; 4 Earth and Environmental Sciences Division, Los Alamos National Laboratory, Los Alamos, NM 87545 USA; 5 Plant Ecology Research Laboratory PERL, School of Architecture, Civil and Environmental Engineering, EPFL, 1015 Lausanne, Switzerland; 6 Functional Plant Ecology, Community Ecology Unit, Swiss Federal Institute for Forest, Snow and Landscape WSL, 1015 Lausanne, Switzerland; 7 Department of Biological Systems Engineering, University of Wisconsin-Madison, Madison, WI 53706 USA; 8 Earth Systems Science Division, Pacific Northwest National Laboratory, Richland, WA 99354 USA

**Keywords:** Drought, Heat, *Ips confusus* (piñon engraver beetle), Monoterpenes, Non-structural carbohydrates

## Abstract

Heat and drought affect plant chemical defenses and thereby plant susceptibility to pests and pathogens. Monoterpenes are of particular importance for conifers as they play critical roles in defense against bark beetles. To date, work seeking to understand the impacts of heat and drought on monoterpenes has primarily focused on young potted seedlings, leaving it unclear how older age classes that are more vulnerable to bark beetles might respond to stress. Furthermore, we lack a clear picture of what carbon resources might be prioritized to support monoterpene synthesis under drought stress. To address this, we measured needle and woody tissue monoterpene concentrations and physiological variables simultaneously from mature piñon pines (*Pinus edulis*) from a unique temperature and drought manipulation field experiment. While heat had no effect on total monoterpene concentrations, trees under combined heat and drought stress exhibited ~ 85% and 35% increases in needle and woody tissue, respectively, over multiple years. Plant physiological variables like maximum photosynthesis each explained less than 10% of the variation in total monoterpenes for both tissue types while starch and glucose + fructose measured 1-month prior explained ~ 45% and 60% of the variation in woody tissue total monoterpene concentrations. Although total monoterpenes increased under combined stress, some key monoterpenes with known roles in bark beetle ecology decreased. These shifts may make trees more favorable for bark beetle attack rather than well defended, which one might conclude if only considering total monoterpene concentrations. Our results point to cumulative and synergistic effects of heat and drought that may reprioritize carbon allocation of specific non-structural carbohydrates toward defense.

**Supplementary Information:**

The online version contains supplementary material available at 10.1007/s00442-021-05058-8.

## Introduction

Drought-induced tree mortality, alone or in conjunction with forest pests and pathogens, has changed ecosystem composition and function across the globe (Allen et al. 2010; Hartmann et al. 2018). Biotic and abiotic stressors are expected to become even more acute in the future (IPCC 2014), emphasizing the need to understand what causes trees to die. Work to date on the mechanisms underlying drought-related tree death has primarily focused on the coupled roles of reduced available carbohydrates and hydraulic conductivity (Anderegg et al. 2012; Hartmann et al. 2013; O’Brien et al. 2014; Sevanto et al. 2014; McDowell et al. 2016; Adams et al. 2017). Yet the carbon and water status of trees is also critical to the synthesis of secondary metabolites, i.e. plant chemical defenses, which in turn, influence the insect population dynamics that also cause tree mortality (Raffa et al. 2008; McDowell et al. 2011; Anderegg et al. 2015). Thus, information on how secondary metabolites change under heat and drought stress provides a critical link between tree stress physiology and the incumbent insect behavior that can ultimately result in tree death. To this end, more field studies are required to better understand the links between well-studied aspects of primary physiology (i.e., photosynthesis) and less understood components, such as non-structural carbohydrates (NSCs) and defense (Ryan et al. 2015). This is especially true considering the majority of our assumptions regarding relationships between these processes and carbon pools are primarily derived from work in young potted seedlings (e.g. Llusia and Penuelas 1998; Turtola et al. 2003; Blanch et al. 2009; Klutsch et al. 2017; Lupke et al. 2017).

Bark beetles (Coleoptera: Curculionidae) and their fungal symbionts are significant biotic disturbance agents affecting coniferous forests (Raffa et al. 2008; Bentz et al. 2010). While climate directly impacts bark beetle population dynamics, fitness is also indirectly affected by climate-induced changes in host quality, namely nutrient availability and oleoresin composition (Raffa and Berryman 1987; Byers 2007; Franceschi et al. 2005). Oleoresin is a mixture of monoterpenes, sesquiterpenes, and diterpene acids produced by trees (Keeling and Bohlmann 2006). Both biotic and abiotic stresses can shift the composition of terpenes within the oleoresin, ultimately determining whether a tree will prove resistant or susceptible to insect pests and microbial pathogens (Keefover-Ring et al. 2016; Trowbridge et al. 2016). The amount of carbon that can be allocated to components within resin depends on both recently assimilated photosynthates as well as stored NSCs (Huang et al. 2019b). Thus, environmental conditions that impact carbohydrate availability, like drought, will undoubtedly have cascading effects on oleoresin production, and in turn, tree defense against biotic agents (Christiansen et al. 1987).

Monoterpenes dominate conifer oleoresin and are of particular interest as they mediate dynamic—but dose- and composition-dependent—relationships among tree hosts, bark beetles, and beetle-associated fungal pathogens (Raffa et al. 2008; Raffa 2013). Generally, high monoterpene concentrations are toxic to bark beetles and their symbionts, as has been demonstrated for both primary aggressive bark beetles, such as mountain pine beetle (*Dendroctonus ponderosae*; Erbilgin et al. 2003), and secondary non-aggressive species like the North American pine engraver (*Ips pini*; Raffa et al. 2005). Both aggressive and non-aggressive bark beetles that rely on aggregation pheromones can also exploit low emissions of monoterpenes for their production. For example, *cis*-verbenol, a minor constituent of the pheromone blend of the pinyon engraver (*Ips confusus*) is produced through the hydroxylation of α-pinene from its host tree, which is also the case for verbenol production by *D. ponderosae* (Chiu et al. 2018; Fisher et al. 2021), Other monoterpenes, such as β-myrcene, may serve as pheromone synergists, but can also be toxic at high levels (Blomquist et al. 2010; Erbilgin et al. 2017). Whether involved in deterrence or aggregation, monoterpene synthesis comes at the expense of carbon that would otherwise be allocated towards processes like growth and storage (Huang et al. 2019a). Furthermore, in response to both abiotic and biotic stress, trees may rely more on NSCs as opposed to newly fixed intermediates (Sevanto and Dickman 2015; Wiley et al. 2016; Roth et al. 2018). The NSCs that are mobilized and the specific defense compounds they support is likely shaped by a combination of past biotic selective pressures and the need to maintain critical physiological functions in the face of current stresses (Cheng et al. 2007; Loreto et al. 2010). Identifying conditions under which chemical defenses are prioritized (or constrained) and trade-offs emerge is critical for calculating risk of mortality by biotic agents (Huang et al. 2020).

**Fig. 1:**
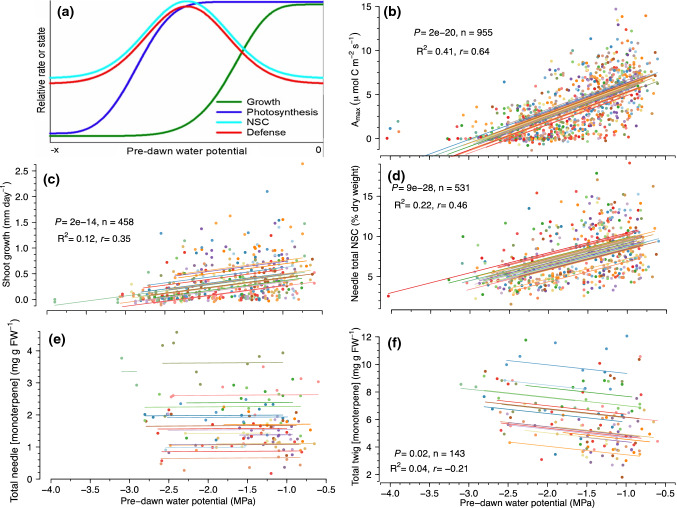
Differences in relative growth rate, net assimilation rate, non-structural carbohydrates (NSCs), and constitutive secondary metabolism across a gradient of resource availability as predicted by **a** the growth-differentiation balance hypothesis (modified from Fig. 1 of Herms and Mattson 1992). **b** Mid-day photosynthesis rate (*A*_max_;), **c** shoot growth rate, **d** total needle NSCs, **e** needle total monoterpene concentrations, and f) woody tissue total monoterpene concentrations from our study are shown as a function of pre-dawn water potential. Each set of data represent all the time points available from 2012 to 2016 for that analysis and relationships between total monoterpenes and pre-dawn water potentials are on a 1-month lag. The best-fit repeated measures regression lines are displayed along with the corresponding equation, *R*^2^, repeated measures correlation coefficient (*r*), *P* value, and sample sizes (*n*). Throughout, different colors refer to different trees

Our conceptual understanding of investment in plant chemical defenses has long been guided by the growth–differentiation balance hypothesis (GDBH) (Loomis 1932; Herms and Mattson 1992; Fig. 1a). The GDBH posits that moderate stress inhibits growth more strongly than it does photosynthesis such that the growth carbon sink strength is dampened and the carbon pool available for secondary metabolism, and thus C allocation towards secondary metabolism itself, is increased. Because drought impedes growth, one would expect an increase in NSC availability and subsequent allocation to monoterpenes as drought stress becomes more severe. There are, however, conflicting reports regarding NSC dynamics during drought (Li et al. 2018); they can decrease (Galiano et al. 2011; McDowell et al. 2013; Woodruff 2014; Dickman et al. 2015), increase (O’Brien et al. 2015), or remain unchanged (Rosas et al. 2013). Furthermore, most studies have focused on patterns of NSC allocation to belowground structures, storage and, osmoregulation (Hartmann and Trumbore 2016; Mackay et al. 2020) with little consideration for chemical defense. Despite the importance of secondary metabolites as carbon sinks, to our knowledge, no studies have experimentally tested the links between drought stress physiology, NSCs, and defense in mature conifers in situ.

Trees are becoming increasingly vulnerable to pests and pathogens under hotter droughts (Allen et al. 2015), yet the interactive effects of heat and drought on monoterpene production in mature trees is largely unknown. Monoterpene production can be quite variable in response to drought (Niinemets 2017). While increased levels of tissue monoterpene concentrations are generally reported in response to drought (e.g., Llusia and Penuelas 1998; Turtola et al. 2003; Blanch et al. 2009; Nowak et al. 2010), these results stem primarily from young potted plants in greenhouse studies. Elevated temperature alone has also been shown to modify terpene metabolism (Penuelas and Munne-Bosch 2005). However, recent studies have provided evidence that drought can override the effects of heat in controlling monoterpene production and emissions (e.g., Trowbridge et al. 2014, 2019), but the observational nature of these studies made it impossible to tease apart the relative contribution of heat and drought. Thus, an enhanced understanding of carbon allocation to defense compounds in response to heat and drought stress, both separately and in concert, would provide crucial insight into mechanisms governing defense production in mature trees.

To elucidate some of the complex relationships between primary and secondary metabolism in situ, we assessed tree water stress status, photosynthesis rate, growth, NSCs, and constitutive monoterpene concentrations across a drought and heat stress gradient. Specifically, we sampled needle and woody tissues from mature piñon pine (*Pinus edulis* (Engelm.)) trees that were part of a large-scale temperature and precipitation manipulation experiment where trees were assigned to one of the following treatments: ambient conditions, heat (~ 5 °C above ambient), drought (~ 45% reduction in precipitation), and combined heat and drought conditions. We address the following research questions: (1) how do prolonged drought and heat stress, separate and combined, alter monoterpene concentrations in different tissues? (2) How do these shifts in total and individual monoterpene concentrations change in relation to primary physiological factors (e.g. growth and photosynthesis) over time? (3) What NSC pools (sucrose, glucose + fructose, starch) are potentially being mobilized to support monoterpene production? We hypothesized that, as predicted by the GDBH, monoterpene concentrations in needles and woody tissues would increase under drought stress and that increased stress/resource limitation by combined heat and drought would result in even greater concentrations. Also, in line with the GDBH, we expected that at drought levels that reduced growth but still supported relatively high rates of photosynthesis, photosynthates would in part support the increased monoterpene concentration. We anticipated that as photosynthesis declined as drought became more severe, we would observe significant relationships between monoterpenes and the soluble sugars sucrose, glucose, and fructose due to a greater reliance on the mobilization of NSCs to support monoterpene biosynthesis. Finally, we rely on previous work regarding the pinyon engraver’s (*Ips confusus*) pheromone production and the effects of monoterpenes on aggressive and non-aggressive bark beetles to discuss how drought-induced shifts in key monoterpenes may impact the pinyon engraver’s host choice and success.

## Materials and methods

### Study site and experimental design

The study was performed near Los Alamos, New Mexico, USA (35.49° N, 106.18° W, elevation 2150 m) at the Los Alamos SUrvival-MOrtality (SUMO) experiment (Adams et al. 2015; Grossiord et al. 2017a; McDowell et al. 2019). The site is located within a piñon–juniper woodland and *Juniperus monosperma* (Engelm.), respectively) near the ponderosa pine (*Pinus ponderosa*) forest ecotone (see Adams et al. (2015) for a full description of the tree community at SUMO). Soils are Hackroy clay loam 40–80 cm above a parent material of volcanic tuff (Soil Survey Staff, Natural Resources Conservation Service, USDA, http://websoilsurvey.nrcs.usda.gov). The local climate is semi-arid (mean annual temperature = 21.1 °C and mean annual precipitation = 401 mm for 1987–2016) and experiences pronounced monsoon rains between July and September.

A manipulative field experiment was established in June 2012 with mature piñon trees (> 10 cm diameter at chest height, mean tree age 56 ± 5 years) assigned to one of five treatments: ambient precipitation and temperature (A), heat stress (~ + 4.8 °C ± 0.3 °C above ambient; H), reduced precipitation (~ 45% rainout; D), and both reduced precipitation and heat stress (~ + 4.8 °C and ~ 45% rainout; HD). There were also chambers with temperatures regulated to match ambient conditions, but plant physiological measurements (e.g. photosynthesis, stomatal conductance, NSC (sugars and starch), respiration, shoot growth, etc.) did not differ between these treatments and the A treatment (Adams et al. 2015) so the A treatment was selected. Mature trees in the heat and combined heat and drought treatments were enclosed in transparent open-top chambers (OTCs) with temperature regulated via heating and cooling units (RJPL Package Heat Pump and Air Conditioner, Rheem Manufacturing Company, Atlanta, GA, USA). The rainout structures, composed of concave plastic troughs on a metal framework (~ 1.3 m above the ground), were designed according to Pangle et al. (2012).

Temperature was monitored at the site and within each OTC at 1 m and 2/3 tree height (CS215 Temperature and Relative Humidity Probe and CR1000 data logger, Campbell Scientific, Logan, UT, USA) to set desired temperature conditions in the chambers. There were a total of 18 chambers with some chambers containing up to five trees. This setup was chosen because some trees grew in clusters and separating them to different chambers was not possible. In some cases, tree replicates for the same treatment were in the same chamber, but there were no more than three trees of the same species (*Pinus edulis* or *Juniperus monosperma*) in any of the chambers. However, for this study we measured four piñons within each treatment. The four piñons sampled within the H + D treatment group were in different chambers, and the only trees that were in the same chamber were two H treatment piñons. A more detailed description of the SUMO experimental design can be found in Adams et al. (2015), Grossiord et al. (2017a), and McDowell et al. (2019).

### Monoterpene sample collection and chemical analysis

Current and 1-year-old piñon pine needles and the distal 10–12 cm of the shoot were collected at chest height from south-facing branches of trees in the A, H, D, and HD treatments (*n* = 4 trees per treatment) across nine sampling dates from 2012 to 2016: one date in 2012 (14 November), two in 2013 (4 April and 18 September), four in 2014 (15 May, 8 July, 5 August, and 9 September), one in 2015 (31 March), and one in 2016 (24 August). Needle and woody tissue samples were immediately flash frozen and stored in liquid nitrogen prior to transport to the lab where they were stored in a − 80 °C freezer before being shipped on dry ice to Montana State University for analysis. Piñon pine tissue processing and analysis followed Trowbridge et al. (2014) (see Supporting Information Methods S1 for more detail).

### Water potential and leaf gas exchange

We measured both mean predawn (ψ_pd_) and midday leaf water potentials (*n* = 2 twigs per tree) for each tree within the A, H, D, and HD treatments (*n* = 4 trees per treatment) using a Scholander pressure chamber (PMS Instruments, Albany, OR). Twigs were excised from sun-exposed portions on the south side of each tree before dawn and between 11:30 and 13:00 on the same days as monoterpene sample collections and monthly over the course of the experiment. Samples were stored in the dark at 20 °C until measurement within 2 h of collection.

We also measured mid-morning photosynthesis (*A*_max_, μmol m^−2^ s^−1^) and stomatal conductance (*g*_s_, mol m^−2^ s^−1^) (LI-6400 infrared gas-exchange analyzer system, Li-Cor, Lincoln, NE, USA) from one current-year or previous-year (depending on whether new needles had emerged) sun-exposed shoot on the south side of each tree. Gas exchange was measured in the morning when the highest stomatal conductance could be expected and was typically measured within one day of water potential measurements. We used the 2 × 3 LED chamber with a reference CO_2_ concentration of 380 ppm, 2000 μmol m^−2^ s^−1^ photosynthetic photon flux density (which is saturating for this species), 20 °C or 25 °C block temperature depending on ambient temperatures, and we kept the relative humidity between 5 and 10% (which was done using the full scrub setting) to mimic typical conditions at our site for all treatments (Grossiord et al. 2017a). Measurements were recorded after steady state gas-exchange rates had been maintained for at least 2 min and chambers were sealed with Qubitac sealing (Channel Technology, Hong Kong) to prevent leakage from the spot where the branchlet enters the chamber. Gas exchange measurements were corrected for projected leaf area, which was measured using an LI-300C area meter (Li-Cor, Lincoln, NE, USA).

### Growth

In 2013, we selected two or three branches on the same four piñon pines within each treatment to measure shoot extension of buds along the main (primary) axis of the branch, and also a paired side branch that diverged from the primary axis 3–5 years prior to measurement (Adams et al. 2015). At the beginning of the growing season (March), we measured bud length with a digital caliper or ruler, then returned at the end of the growing season (October or November) to measure final branch length. At the beginning of each subsequent year of measurement (2014–2016), if main axis buds were dead, damaged, or missing, we selected replacement branches such that there were always at least two measurement branches on each tree. Because shoot growth increments can be easily distinguished and dated in piñon pine, we also measured the shoot lengths of the prior three years at the time of the first branch selection in 2013. This resulted in a 7-year record of annual shoot growth, from 2010 to 2016. Additionally, in 2013 and 2014, we measured shoot extension periodically during the growing season, on average every 18 days in 2013 and every 26 days in 2014. From these measurements, we calculated daily growth rates of shoot extension assuming steady growth rate throughout the period.

### Non-structural carbohydrates

Foliar and twig samples for NSC analysis were collected between 11:30 and 13:00 from the same four trees per treatment four times during each growing season (usually April, June, August, October). Samples for NSC analyses were collected concurrently with monoterpene samples for six of nine monoterpene sampling dates; the other three dates (May, July, and August of 2014) had samples for NSCs collected within one month of the monoterpene tissue samples. Once collected, samples for NSCs were frozen in liquid N_2_ at collection, transported to Los Alamos National Laboratory on dry ice, stored at − 70 °C until microwaved for 5 min at 800 W, and then dried at 65 °C for 48 h. We pre-ground samples in a Wiley mill (Thomas Scientific, Swedesboro, NJ, USA) then ground all samples into a fine powder using a ball mill (VWR, Radnor, PA, USA). We measured soluble sugars (glucose, fructose, and sucrose) and starch concentrations following Dickman et al. (2015), which was modified from Hoch et al. (2002). We used water extraction, enzymatic starch digestion (with amyloglucosidase), and enzymatic sugar quantification (with phosphoglucose isomerase, invertase, glucose hexokinase, and glucose-6-P dehydrogenase). More details are provided in Adams et al., (2015). Comparison of NSC measurements among laboratories and protocols can be problematic (Quentin et al. 2015, Landhäusser et al. 2018), but we are confident in comparison among treatments in this study as all measurements were made in the same laboratory using the same protocol. We report concentrations as percentage of dry weight.

### Data analysis

Mixed model analyses were performed using SAS statistical software 9.4 (SAS Institute, Cary, North Carolina, USA) to compare monoterpene chemistry, *A*_max_, and growth data between treatments. All data were confirmed to meet the assumption of normality. For individual monoterpene compounds, we performed separate single-factor ANOVAs (SAS: PROC MIXED) and the sum of all measured monoterpene concentrations for each tissue type, hereafter called ‘total’. For all mixed models, we included treatment and time as the fixed factors and tree (nested in treatment) as the random factor to account for our repeated measures approach. Tukey’s post hoc analyses were employed if main effects were significant. It is prudent to note here that the level of drought imposed on the trees did not significantly affect the needle tissue water content, and thus the fresh weight:dry weight (FW:DW) coefficient of trees among treatments (Supporting Information Fig. S1A). Ambient trees had a significantly higher FW:DW coefficient relative to trees in all other treatments, but the effect size was very low (Supporting Information Fig. S1B). Because fresh and dry weight monoterpene data for both tissue types provided the same statistical results, we present all monoterpene concentration data here on a fresh weight (FW) basis (Trowbridge et al. 2014, 2019). This also avoids introducing unnecessary error by converting all values to a dry weight using a conversion factor when chemistry was originally extracted from fresh mass.

To test correlations and linear models we used R version 3.5.2 (R Core Team 2018). We used repeated measures correlation analysis via the *rmcorr* package (Bakdash and Marusich 2017) to assess potential relationships between needle and woody tissue monoterpene concentrations (totals and individual compounds), NSCs, and what we call ‘primary’ physiological variables (e.g. shoot growth rate, ψ_*pd*_, *A*_max_, *g*_s_, midday water potential, etc.) and performed Bonferroni corrections to account for multiple pairwise comparisons. Because physiological variables and secondary chemistry were measured at different frequencies (e.g., *A*_max_, ψ_pd_, and NSCs were measured more frequently than monoterpenes), analyses that correlate primary physiology with secondary chemistry contain less data than those testing relationships among primary physiological variables. To explore potential lag responses between NSCs and total monoterpenes, we compared correlations using NSC data from the month in which monoterpenes were measured and also 1 and 2 months prior. We performed compositional analyses to determine differences in needle and woody tissue monoterpene composition among treatment, time, and their interaction using permutational multivariate ANOVA (Anderson et al. 2014; Oksanen et al. 2019). To do so, we used the ‘vegdist’ function in the ‘vegan’ package in R (Oksanen et al. 2019) to calculate dissimilarities among samples using the Bray–Curtis metric with tree ID as the strata to control for repeated measures on the same tree. To visualize needle and woody tissue monoterpene composition among treatment averaged over nine sample periods, we used non-metric multidimensional scaling (NMDS) and plotted the first two axes.

## Results

### Effects of heat and drought stress on gas exchange, growth, and NSCs

Across the sampling periods, there was a significant effect of time and the interaction of treatment and time on *A*_max_ (*F*_8,92_ = 33.60, *P* < 0.0001 and *F*_24, 92_ = 1.72, *P* = 0.035, respectively). Over the course of the experiment, *A*_max_ in target trees was markedly lower in combined heat and drought trees (3.4 ± 0.4 μmol m^−2^ s^−1^) relative to trees in both ambient (5.6 ± 0.4 μmol m^−2^ s^−1^, *P* = 0.002) and heat treatments (4.7 ± 0.4 μmol m^−2^ s^−1^, *P* = 0.02). *A*_max_ in trees experiencing drought (4.1 ± 0.4 μmol m^−2^ s^−1^) was no different from combined heat and drought trees and, while lower than ambient, were not different from heated trees alone. As expected, *A*_max_ was positively correlated with predawn leaf water potential (Fig. 1b), meaning that assimilation was reduced with increasing drought stress.

Primary shoot growth rate (mm day^−1^) was positively correlated with ψ_pd_ (*r* = 0.35, *R*^2^ = 0.12, *n* = 458, *P* < 0.0001) meaning it decreased with increasing drought stress (Fig. 1c). The effect of year on annual growth was significant (*F*_6,66_ = 25.89, *P* < 0.0001), and while there was no treatment effect, there was a significant treatment × year interaction (*F*_18,66_ = 2.70, *P* = 0.0018) (Supporting Information Fig. S2). Within years, there were significant differences in annual primary growth by treatment in 2013 and 2015 where the only differences were reduced growth in heat, drought, and combined heat and drought trees relative to controls (2013: *F*_3,11_ = 8.70, *P* = 0.003 and 2015: *F*_3,11_ = 3.92, *P* = 0.04); Supporting Information Fig. S2).

Total NSCs in needles were positively correlated with ψ_pd_ (*r* = 0.46, *R*^2^ = 0.22, *P* < 0.0001, *n* = 531, Fig. 1d), driven primarily by positive correlations with needle starch (*r* = 0.44, *R*^2^ = 0.19, *P* < 0.0001, *n* = 531, Table 1). There was no correlation between total twig NSCs and ψ_pd_ (Table 1) despite negative correlations with twig sucrose (*r* =  − 0.27, *R*^2^ = 0.07, *P* < 0.0001, *n* = 531) and twig glucose + fructose (*r* =  − 0.33, *R*^2^ = 0.11, *P* < 0.0001, *n* = 531, Table 1). In both tissues, total monoterpene concentrations were uncorrelated (needles) or poorly correlated (twigs) with ψ_pd_ (Fig. 1e and f), which explained only 4% of the variation.

**Table 1:** Correlations between non-structural carbohydrates and pre-dawn water potential from *Pinus edulis* twigs

Tree organ	Total NSCs	Starch	Sucrose	Glucose + fructose
Leaf	**0.46 (< 2.2 × 10**^**−16**^**)**	**0.44 (< 2.2 × 10**^**−16**^**)**	0.10 (0.019)	0.11 (0.011)
Twig	− 0.03 (0.49)	0.11 (0.013)	** − 0.27 (7 × 10**^**−10**^**)**	** − 0.33 (1 × 10**^**−14**^**)**
Bole	** − 0.35 (0.0006)**	− 0.03 (0.77)	** − 0.57 (3 × 10**^**−9**^**)**	** − 0.66 (1 × 10**^**−12**^**)**
Root	− 0.25 (0.07)	0.019 (0.89)	** − 0.50 (0.0001)**	** − 0.61 (1 × 10**^**−6**^**)**

Repeated measures *r* correlation coefficient (*P* value) is displayed and significant correlations after applying the Bonferroni correction for multiple comparisons (α < 0.0031) are indicated in bold. Negative correlation coefficients represent increasing values of NSCs with increasing drought stress (more negative pre-dawn water potential values) while positive coefficients represent decreasing values of NSCs with increased drought stress

### Effects of heat and drought on total monoterpene concentrations

Sixteen monoterpenes were identified in the needle and wood samples: (−)-α-pinene, ( +)-α-pinene, tricyclene, (−)-camphene, (+)-camphene, β-myrcene, (+)-β-pinene, (−)-β-pinene, δ-3-carene, S-(−)-limonene, R-( +)-limonene, β-ocimene, β-phellandrene, γ-terpinene, and terpinolene, as well as bornyl acetate (C_12_H_20_O_2_), a monoterpene ester. Tricyclene was only identified in needle samples while β-ocimene and (+)-β-pinene were only identified in samples from woody tissues. Hereafter, “total monoterpenes” and “total monoterpene concentrations” are used to represent the sum of all the aforementioned compounds present in each tissue type.

**Fig. 2:**
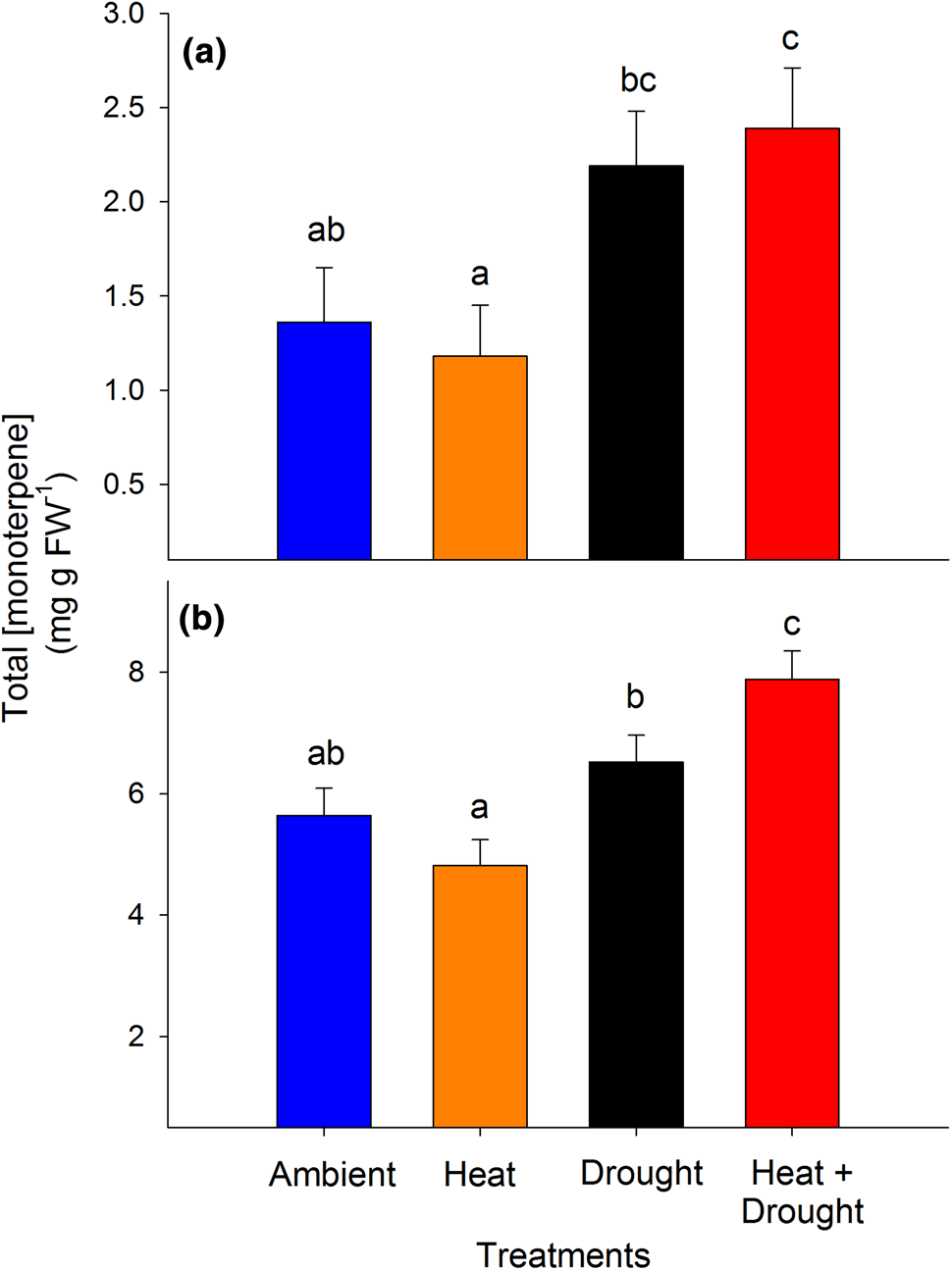
Total monoterpene compound concentrations (mg g FW-1) in **a** *Pinus edulis* needles and **b** woody tissue across the four treatments averaged over nine sampling periods from 2012 to 2016. Bars are means ± SEM and significant differences between treatments is expressed using differing lowercase letters (α < 0.05)

**Fig. 3:**
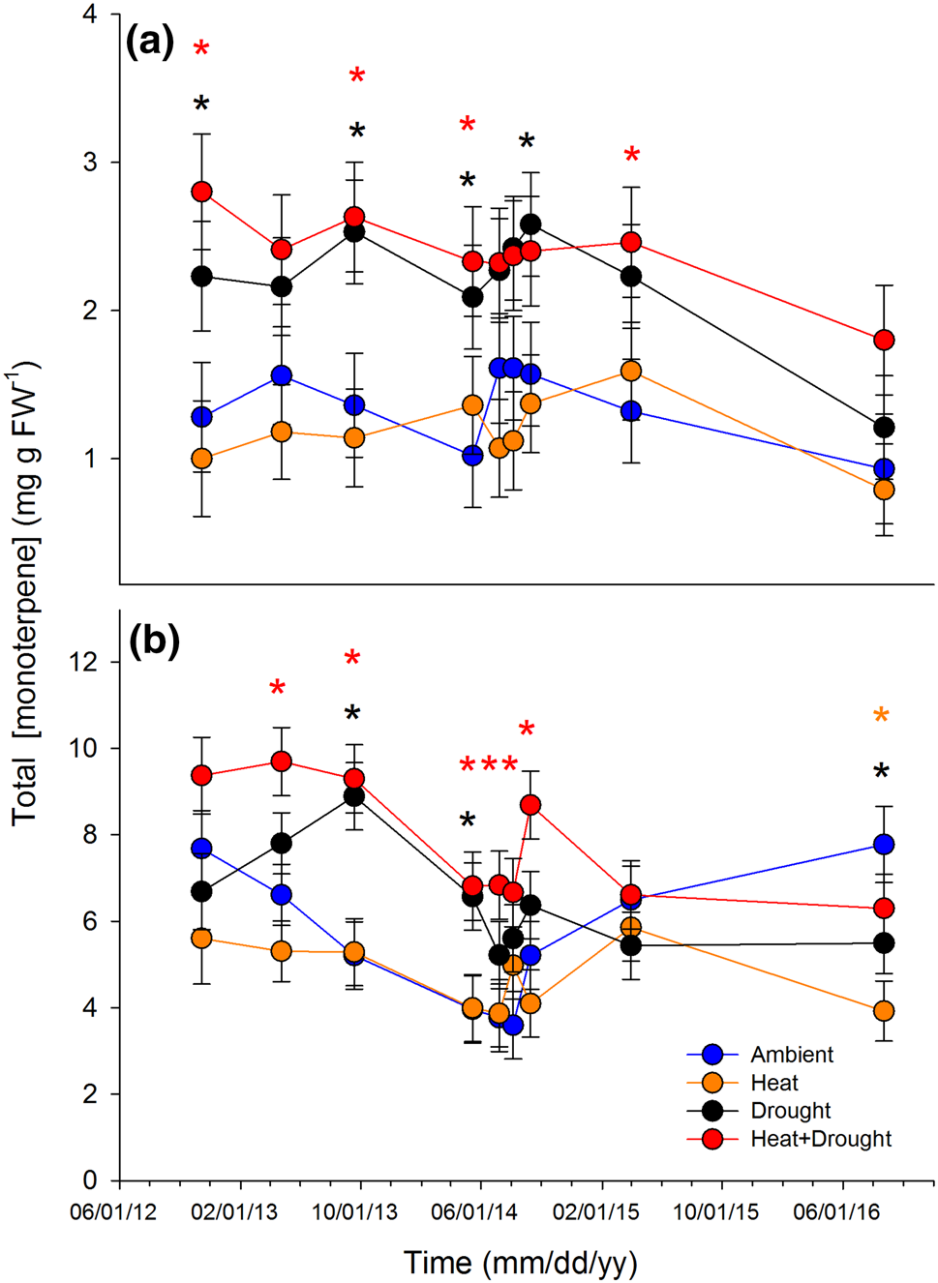
Time series data representing means (± SEM) of total monoterpene concentrations (mg g FW^−1^) **a** needles and **b** woody tissue for *Pinus edulis* exposed to the four treatments across nine sampling dates from 2012 to 2016. Totals were calculated by summing all identified monoterpenes for each tree (*n* = 14 for needle tissue; *n* = 16 for woody tissue) within each treatment (*n* = 4) during each sampling period. Different colored asterisks represent a significant difference from ambient (⍺ < 0.05) for that treatment

Total monoterpenes in trees experiencing combined heat and drought were nearly 85% higher in needles (Fig. 2a) and ~ 35% higher in woody tissues (Fig. 2b) relative to trees in the ambient (needles: *P* = 0.03 and woody tissue: *P* = 0.02) and heat stress (needles: *P* = 0.01 and woody tissue: *P* = 0.0015) treatment groups. In both tissues, trees in the drought treatment alone exhibited higher total needle monoterpene concentrations relative to trees from the heat treatment group (*P* = 0.02; Fig. 2), while monoterpene levels in the heat trees were not different from trees exposed to ambient conditions. Unlike in the needles, woody tissue monoterpene concentrations in the combined heat and drought trees were higher than those in the drought alone treatment (*P* = 0.05). These trends were the same when concentrations were analyzed on a dry weight basis (Supporting Information Fig. S3). In both tissues, treatment and time had significant effects on total monoterpenes (needles: Fig. 3a, treatment: *F*_3,16_ = 4.13, *P* = 0.02; time: *F*_8,90_ = 5.52, *P* < 0.0001 and woody tissue: Fig. 3b, treatment: *F*_3,15_ = 8.38, *P* = 0.002; time: *F*_8,94_ = 7.17, *P* < 0.0001). There was also a treatment × time interaction for total concentrations in the woody tissue (Fig. 3b, treatment × time: *F*_24,94_ = 2.15, *P* = 0.005). The timing of treatment effects on total monoterpenes, however, differed between tissue types. Responses in the needles were evident upon first measurement (Fig. 3a), where concentrations in the drought and the combined heat and drought trees remained elevated relative to ambient trees until 2015. In contrast, differences between the drought and combined heat and drought treatments relative to ambient were not apparent within woody tissues for approximately one year following the initiation of the treatments (Fig. 3b).

### Relationships between total monoterpenes and physiological variables

**Fig. 4:**
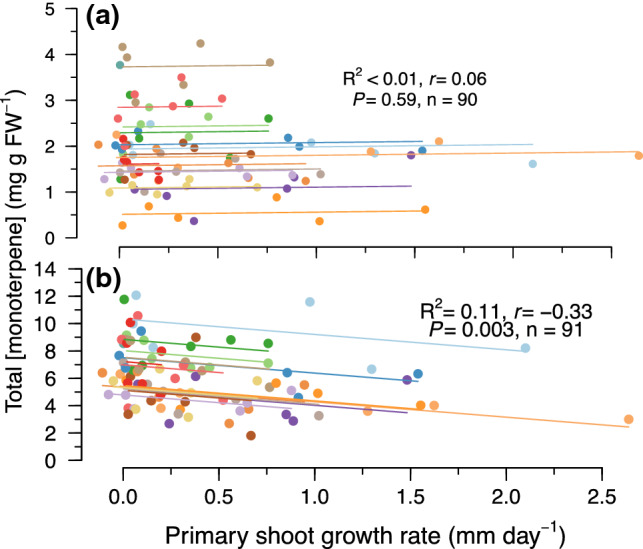
Repeated measures correlations between total monoterpene concentrations (mg g FW^−1^) and primary shoot growth rates (mm day^−1^) in **a** needles and **b** woody tissue in *Pinus edulis* from the four treatments for six sampling periods from 2013 to 2014. Solid lines represent the best-fit repeated measures regression line for the data and the linear equations, *P* values, repeated measures correlation coefficients (*r*), and sample sizes (*n*) are presented

**Fig. 5:**
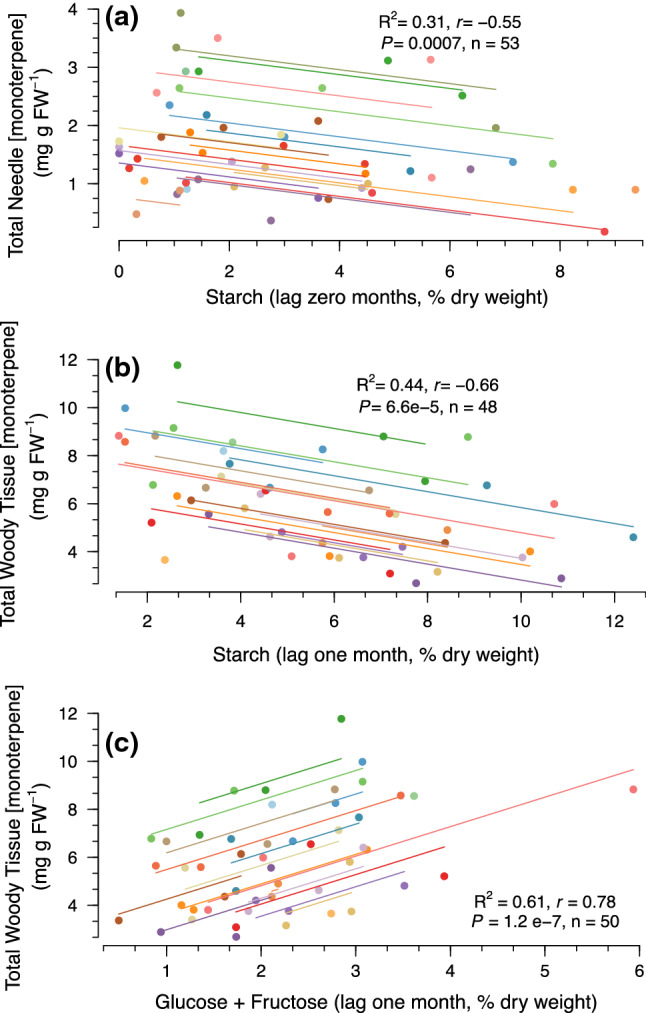
The relationships between **a** needle total monoterpene concentrations (mg g FW^−1^) and starch content (% dry weight) measured during the same month, and between woody tissue total monoterpene concentration and **b** starch and **c** glucose + fructose on a 1-month time lag. Solid lines represent the best-fit repeated measures regression line for the data and the linear equations, *P*-values, repeated measures correlation coefficients (*r*), and sample sizes (*n*) are presented

Total monoterpenes in the leaf tissue were not correlated to mid-day water potential, *A*_max_, *g*_s_, or primary shoot growth rates (data not shown for mid-day water potential, *A*_max_, *g*_s_, but see Fig. 4a for shoot growth rate). Unlike the leaf tissue, however, total monoterpenes in the woody tissue were inversely correlated to primary shoot growth rate, which explained 11% of the variation (Fig. 4b). Total monoterpenes in the needles exhibited a negative correlation with starch as well as total NSCs measured that same month (*r* =  − 0.55, *R*^2^ = 0.31, *P* = 0.0007, *n* = 53, Fig. 5a, Table 2) and were not correlated to other NSCs at any time point (Table 2). In contrast, total monoterpenes in the woody tissue were negatively correlated with starch from the previous month (*r* =  − 0.66, *R*^2^ = 0.44, *P* =  < 0.0001, *n* = 48, Fig. 5b, Table 2) and positively correlated with the sum of glucose and fructose from the previous month (*r* = 0.78, R^2^ = 0.61, *P* < 0.0001, *n* = 50, Fig. 5c, Table 2); each variable explained 44% and 61% of the variation, respectively.

**Table 2:** Correlations between leaf and twig total monoterpene concentrations and non-structural carbohydrates in *Pinus edulis*

Tissue	Lag	Total NSCs	Starch	Sucrose	Glucose + fructose	Total sugars
Leaf	0	** − 0.55 (0.0007)**	** − 0.55 (0.0007)**	− 0.29 (0.09)	− 0.07 (0.70)	− 0.48 (0.004)
Leaf	1	0.06 (0.75)	0.08 (0.65)	− 0.23 (0.23)	0.14 (0.45)	− 0.03 (0.87)
Leaf	2	0.38 (0.16)	0.51 (0.051)	− 0.19 (0.50)	− 0.14 (0.61)	− 0.25 (0.37)
Twig	0	− 0.10 (0.56)	− 0.4 (0.43)	− 0.21 (0.23)	0.33 (0.04)	0.05 (0.76)
Twig	1	− 0.48 (0.007)	** − 0.66 (6 × 10**^**−5**^**)**	− 0.02 (0.93)	**0.78 (1 × 10**^**−7**^**)**	**0.54 (0.002)**
Twig	2	0.014 (0.96)	0.06 (0.84)	− 0.39 (0.19)	0.18 (0.55)	− 0.09 (0.77)

Repeated measures *r* correlation coefficient (*P* value) is displayed and significant correlations after applying the Bonferroni correction for multiple comparisons (α < 0.0017) are indicated in bold. Negative correlation coefficients represent increasing values of NSCs with increasing drought stress (more negative pre-dawn water potential values) while positive coefficients represent decreasing values of NSCs with increased drought stress

### Effects of heat and drought on monoterpene composition and individual compounds

**Fig. 6:**
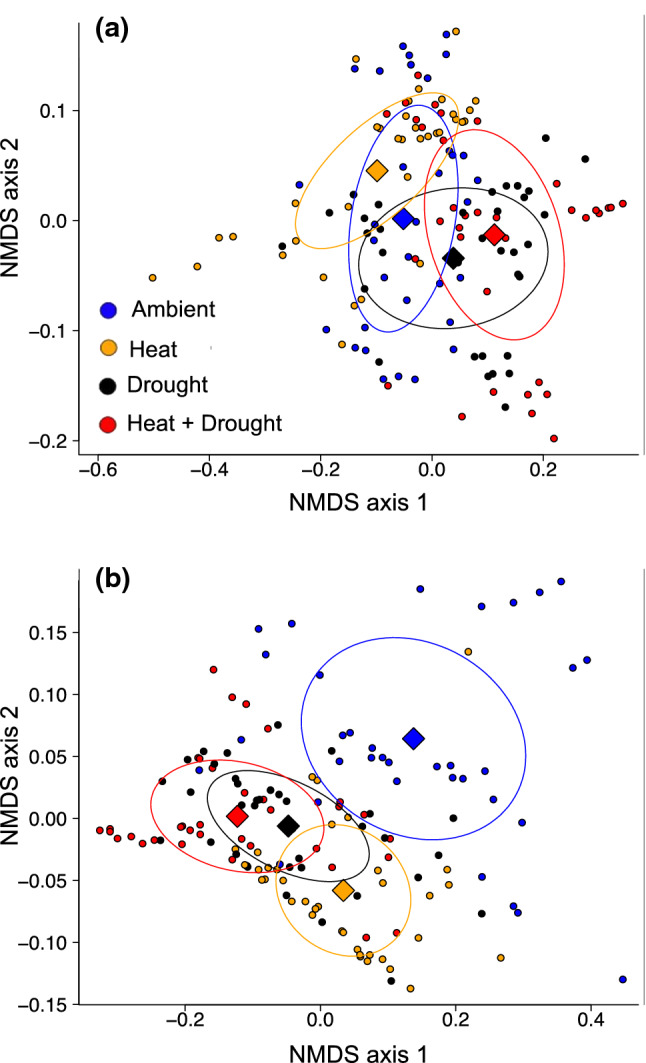
Nonmetric multidimensional scaling (NMDS) of treatment effect on monoterpene composition in *Pinus edulis* **a** needles and **b** woody tissue across the four treatments averaged over nine sampling periods (2012–2016). Ellipses encircle the centroids (diamonds) and the relative monoterpene composition of all the tree individuals from the same treatment

In the ambient treatment, (−)-α-pinene, (+)-α-pinene, β-phellandrene, and (−)-β-pinene were the four most common monoterpenes and made up ~ 84% of the total monoterpene composition in the needle tissue (Supporting Information Table S1) while (+)-α-pinene, δ-3-carene, and β-myrcene made up ~ 87% of the total monoterpene composition in the woody tissue (Supporting Information Table S2). Both treatment and time shifted the overall composition of monoterpenes in the needles (Supporting Information Table S3), but only the chemical profile of heat trees was different than ambient (Fig. 6a and Supporting Information Fig. S4a). Treatment and time also had significant effects on the overall composition of woody tissue monoterpenes (Supporting Information Table S3), but in contrast with needles, all treatment trees (heat, drought, and combined stress) demonstrated more distinctive monoterpene profiles relative to ambient (Fig. 6b and Supporting Information Fig. S4b).

**Fig. 7:**
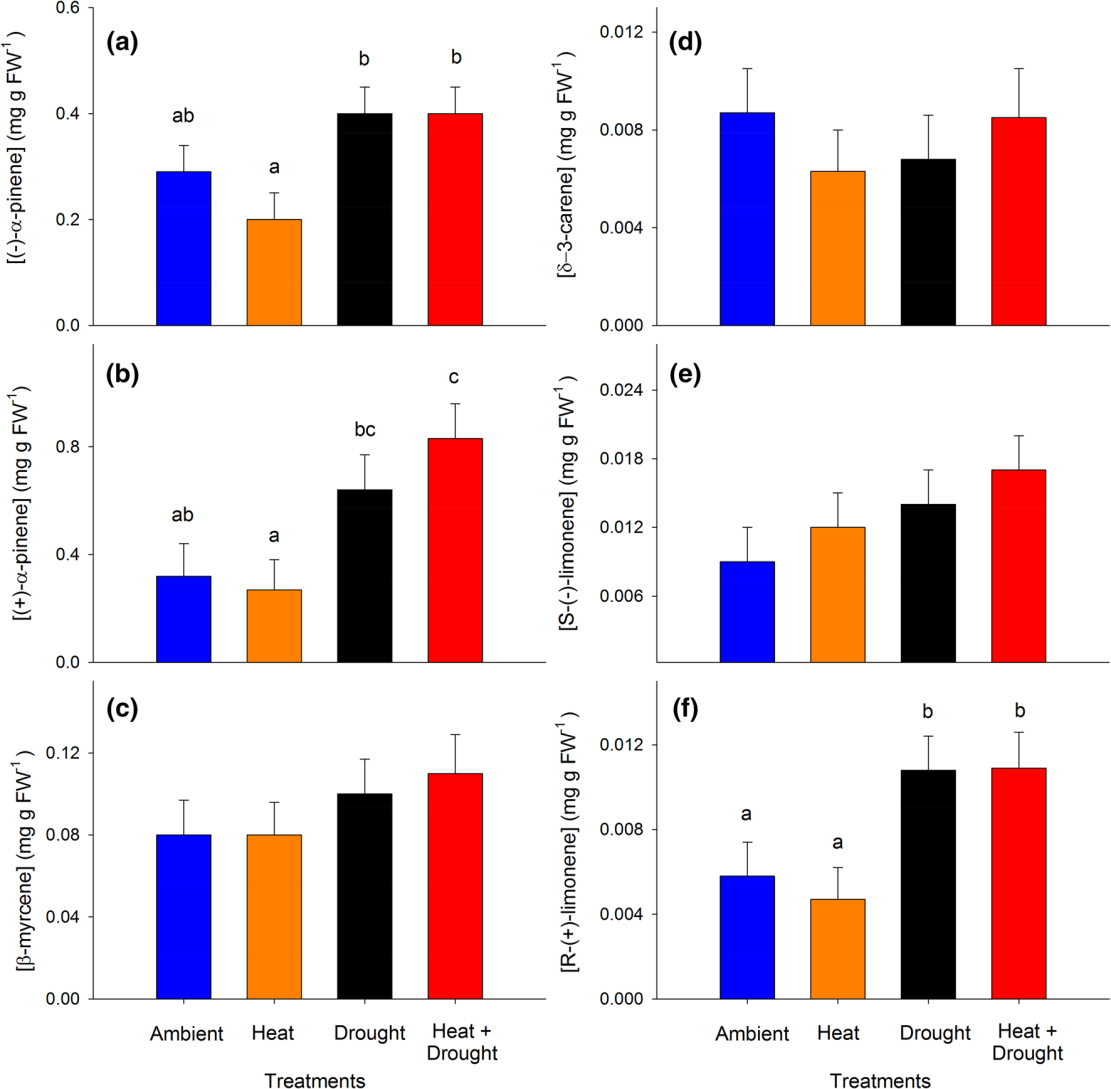
Mean individual monoterpene concentrations (mg g^−1^ FW) of six compounds found in *Pinus edulis* current and one-year old needle tissue across the four treatments averaged over nine sampling periods (2012–2016). **a** (−)-α-Pinene, **b** (+)-α -pinene, **c** β -myrcene, **d** δ-3-carene, **e** S-(-)-limonene, f) R-( +)-limonene. Compounds were chosen to highlight the variation in responses across treatments and tissues. Bars represent means ± SEM and significance between treatments is expressed using different lowercase letters (α < 0.05)

The treatments had significant effects on six of the fourteen compounds identified in the needles (Table 3). The most notable change in individual compound concentration involved an approximately twofold increase in (−)-α-pinene and (+)-α-pinene under combined heat and drought stress compared to heat alone (Fig. 7a and b). Similarly, R-(+)-limonene increased threefold in response to drought and combined heat and drought (Fig. 7f); however, several other compounds (including its enantiomer) were notably unaffected by the treatments (e.g., β-myrcene, δ-3-carene, and *S*-(−)-limonene concentrations, Fig. 7c, d, and e, respectively). NSC content never explained more than 12% of the variation in individual compound concentrations in response to stress, except γ-terpinene in twigs where glucose + fructose explained 18% of the variance (Supporting Information Table S4).

**Table 3:** Mean individual monoterpene concentrations from *Pinus edulis* needles and twigs

Compound	Needles				Woody tissue			
	Ambient	Heat	Drought	Heat + drought	Ambient	Heat	Drought	Heat + drought
(−)-α-Pinene	285 ± 49^ab^	197 ± 46^a^	398 ± 49^b^	393 ± 54^b^	278 ± 41^a^	287 ± 39^a^	414 ± 40^b^	451 ± 44^b^
(+)-α-Pinene	324 ± 118^ab^	274 ± 110^a^	640 ± 118^bc^	830 ± 130^c^	3547 ± 0.518^a^	3748 ± 489^a^	5050 ± 511^ab^	6334 ± 561^b^
Tricyclene	3.4 ± 1.3^a^	4.2 ± 1.2^a^	5.0 ± 1.3^a^	6.2 ± 1.5^a^	NA	NA	NA	NA
(−)-Camphene	0.7 ± 0.2^a^	0.6 ± 0.2^a^	0.6 ± 0.2^a^	0.6 ± 0.2^a^	12 ± 1.5^a^	13 ± 1.5^a^	17 ± 1.5^ab^	22 ± 1.6^b^
(+)-Camphene	0.09 ± 0.02^ab^	0.07 ± 0.02^a^	0.12 ± 0.02^bc^	0.14 ± 0.02 cd	23 ± 3.5^a^	24 ± 3.3^a^	33 ± 3.5^ab^	41 ± 3.8^b^
β-Myrcene	75 ± 17^a^	79 ± 16^a^	95 ± 17^a^	112 ± 19^a^	592 ± 128^a^	272 ± 120^a^	521 ± 126^a^	524 ± 139^a^
(+)-β-Pinene	NA	NA	NA	NA	27 ± 4.9^a^	23 ± 4.6^a^	31 ± 4.8^a^	36 ± 5.3^a^
(−)-β-Pinene	222 ± 57^ac^	122 ± 53^a^	407 ± 57^b^	304 ± 63^bc^	105 ± 44^a^	69 ± 41^a^	164 ± 44^a^	141 ± 49^a^
δ-3-Carene	8.8 ± 1.8^a^	6.3 ± 1.7^a^	6.8 ± 1.8^a^	8.5 ± 2.0^a^	835 ± 162^a^	258 ± 150^b^	95 ± 162^b^	91 ± 180^b^
S-(−)-Limonene	10 ± 3^a^	12 ± 3^a^	14 ± 3^a^	17 ± 3^a^	10 ± 0.6^a^	6.7 ± 0.6^b^	9 ± 0.6^a^	11 ± 0.6^a^
R-(+)-Limonene	5.8 ± 1.6^a^	4.7 ± 1.5^a^	10.8 ± 1.6^b^	10.9 ± 1.7^b^	18 ± 2.5^a^	18 ± 2.4^a^	25 ± 2.5^ab^	32 ± 2.7^b^
β-Ocimene	NA	NA	NA	NA	36 ± 8.0^a^	48 ± 7.5^a^	42 ± 7.9^a^	43 ± 8.8^a^
β-Phellandrene	312 ± 154^a^	389 ± 142^a^	513 ± 154^a^	625 ± 171^a^	49 ± 14^a^	28 ± 13^a^	51 ± 14^a^	45 ± 15^a^
γ-Terpinene	1.2 ± 0.5^a^	1.5 ± 0.4^a^	1.4 ± 0.5^a^	2.2 ± 0.5^a^	11 ± 2.1^a^	2 ± 2.1^b^	5 ± 2.2^ab^	5 ± 2.4^ab^
Terpinolene	3.6 ± 0.6^a^	3.0 ± 0.6^a^	4.5 ± 0.6^ab^	5.7 ± 0.6^b^	95 ± 13^a^	28 ± 12^b^	39 ± 12^b^	32 ± 14^b^
Bornyl acetate	117 ± 39^a^	86 ± 36^a^	105 ± 39^a^	74 ± 24^a^	47 ± 6.6^ac^	43 ± 6.2^a^	64 ± 6.5^b^	75 ± 7.1^bc^

Differences in lower case letters represent significant differences in concentrations between treatments for each compound and tissue type are designated by (α < 0.05)

**Fig. 8:**
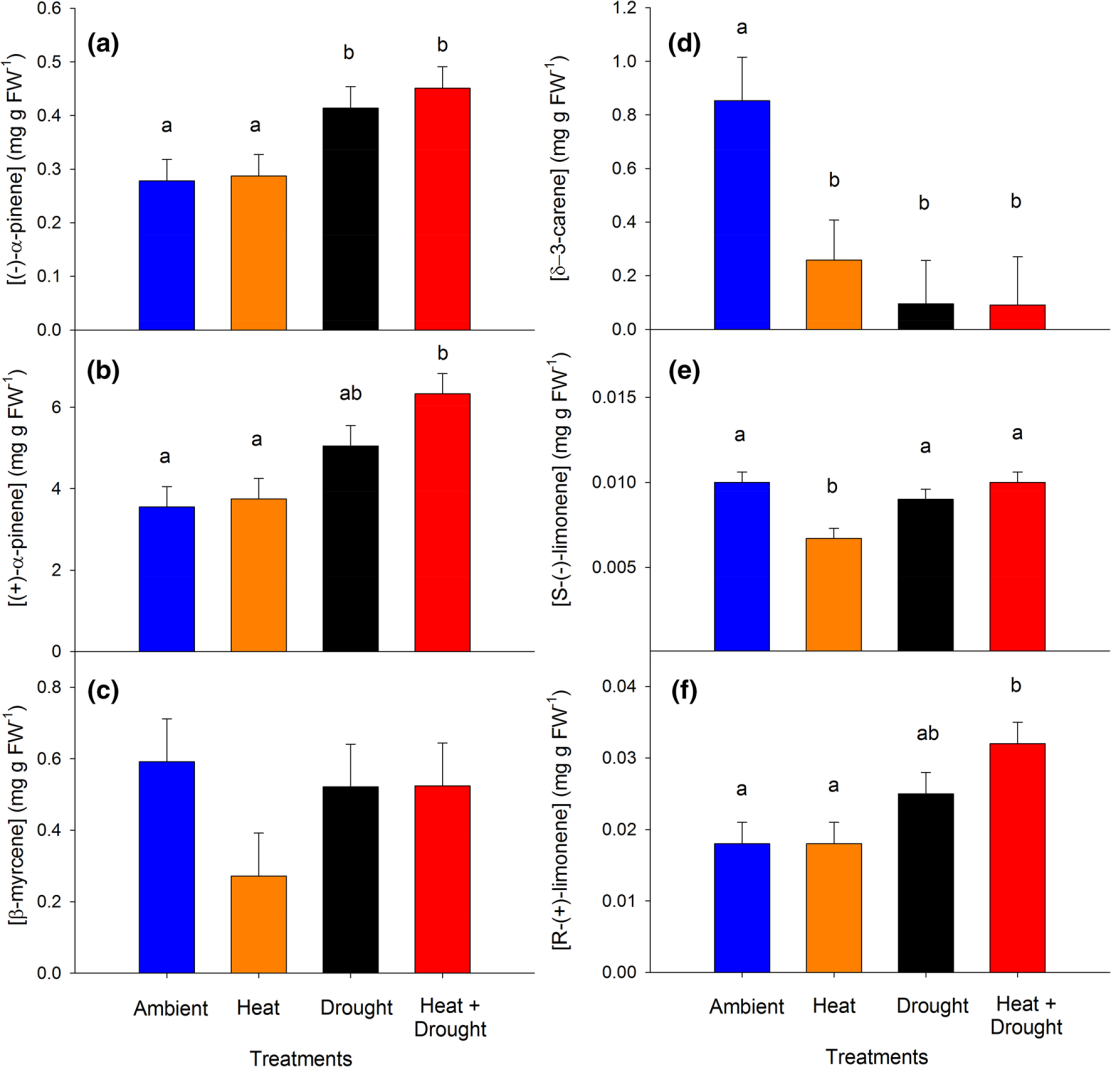
Monoterpene concentrations (mg g^−1^ FW) of six compounds in *Pinus edulis* woody tissue across the four treatments averaged over nine sampling periods (2012–2016). **a** (−)-α-Pinene, **b** (+)-α -pinene, **c** β -myrcene, **d** δ-3-carene, **e** S-(−)-limonene, **f** R-(+)-limonene. Compounds were chosen to highlight the variation in responses across treatments and tissues. Bars are means ± SEM and significance between treatments is expressed using different lowercase letters (α < 0.05)

Treatments had significant effects on nine of the fifteen compounds identified in the woody tissues (Table 3). Notable changes in individual compound concentrations included ~ 62% and 42% increases in (−)-α-pinene (Fig. 8a) and (+)-α-pinene (Fig. 8b), respectively, in trees from the combined heat and drought treatment relative to ambient. Combined heat and drought trees also exhibited ~ 60% increase in bornyl acetate (Table 3) and ~ 80% increases in (+)-R-limonene (Fig. 8f) and both enantiomers of camphene (Table 3). Like the pattern observed in needles, there was no treatment effect on β-myrcene concentrations (Fig. 8c). However, unlike the needles, heat significantly decreased levels of a number of woody tissue compounds relative to ambient including *S*-(−)-limonene (Fig. 8e), γ-terpinene, and terpinolene (Table 3). Heat alone also led to a threefold decrease in δ-3-carene, and interestingly, drought and combined heat and drought caused a tenfold decrease relative to ambient (Fig. 8d). Several individual monoterpene compounds in the woody tissues were significantly correlated with glucose + fructose but not with starch or sucrose (Table 4). Similar to needle tissues, NSC content never explained more than 14% of the variation in individual compound concentrations in response to stress (Table 4).

**Table 4:** Correlations between individual monoterpene concentrations and non-structural carbohydrates in twigs of *Pinus edulis*

Compound	Starch	Sucrose	Glucose + fructose
(−)-α-Pinene	− 0.24 (0.016)	− 0.09 (0.39)	**0.38 (9 × 10**^**−5**^**)**
(+)-α-Pinene	− 0.29 (0.003)	− 0.04 (0.69)	**0.31 (0.001)**
(−)-Camphene	− 0.24 (0.017)	− 0.06 (0.53)	0.29 (0.002)
(+)-Camphene	− 0.30 (0.002)	− 0.06 (0.54)	**0.33 (0.0007)**
β-Myrcene	− 0.25 (0.012)	− 0.002 (0.98)	0.25 (0.009)
(+)-β-Pinene	− 0.27 (0.007)	− 0.10 (0.32)	0.26 (0.008)
(−)-β-Pinene	0.09 (0.40)	− 0.07 (0.48)	− 0.002 (0.99)
δ-3-Carene	− 0.19 (0.06)	− 0.06 (0.51)	0.29 (0.003)
(−)-Limonene	− 0.23 (0.02)	− 0.11 (0.27)	0.29 (0.003)
(+)-Limonene	− 0.26 (0.01)	− 0.04 (0.67)	**0.36 (0.0002)**
β-Ocimene	− 0.17 (0.08)	− 0.08 (0.42)	0.18 (0.06)
β-Phellandrene	− 0.07 (0.49)	− 0.02 (0.81)	0.05 (0.61)
g-Terpinene	− 0.008 (0.93)	0.07 (0.48)	0.04 (0.71)
Terpinolene	− 0.23 (0.02)	− 0.01 (0.92)	0.28 (0.004)
Bornyl acetate	− 0.21 (0.03)	0.01 (0.90)	0.29 (0.003)

Repeated measures *r* correlation coefficient (*P* value) is displayed and significant correlations after applying the Bonferroni correction for multiple comparisons (α < 0.0011) are indicated in bold. Negative correlation coefficients represent increasing values of NSCs with increasing drought stress (more negative pre-dawn water potential values) while positive coefficients represent decreasing values of NSCs with increased drought stress

## Discussion

We leveraged a unique manipulative temperature and drought experiment to explore relationships between primary and secondary metabolism in mature piñon pine across a water-stress gradient in situ. The data support our hypothesis that, as predicted by the GDBH, monoterpene concentrations would increase in both tissues under drought stress (Fig. 2); however, increased stress/resource limitation by combined heat and drought only resulted in greater monoterpene concentrations (relative to drought alone) in the woody tissues (Fig. 2b). Not only were woody tissue total monoterpene concentrations in trees under combined heat and drought significantly higher than levels observed in both heat-stressed and ambient trees, but this increase was sustained over multiple growing seasons (Fig. 3b). We also expected a relationship between monoterpenes and the soluble sugars sucrose, glucose, and fructose under increased stress due to a greater reliance on NSC mobilization to support monoterpene biosynthesis as photosynthesis declines. Maximum photosynthetic rates were suppressed under combined heat and drought stress, yet woody tissue monoterpene concentrations were elevated. Our data suggest these elevated woody tissue monoterpene levels in the face of low *A*_max_ rates are supported in part through fructose and glucose mobilization via starch hydrolysis (Fig. 5) on a 1-month lag. Furthermore, individual monoterpenes were affected by heat and drought in different ways (Table 3) highlighting the need to consider both tissue source and specific compounds as well as lags in effects when determining context for how and when monoterpene synthesis is regulated and the consequences for species interactions.

### Physiological variables and total monoterpenes

Consistent with the GDBH and previous work in conifers (e.g., Llusia and Penuelas 1998; Turtola et al. 2003; Blanch et al. 2009), total monoterpene concentrations in both tissues increased under resource-limiting conditions (Fig. 2). These elevated levels under prolonged drought stress suggests that physiological stress levels required to initiate a decline in total monoterpene production in piñon pine likely occur at lower water potentials than observed, i.e. <  − 3 MPa, which was not reached during our study due to access to deep soil water and amelioration of the imposed water limitation (Grossiord et al. 2017b; McDowell et al. 2019). While growth rates were correlated to woody tissue total monoterpenes, they did not explain more than 11% of variation in concentrations (Fig. 4b) offering very little mechanistic explanatory power. Rather, elevated total monoterpene concentrations in the combined heat and drought treatments—with lower assimilation rates and reduced growth sink strength (Supporting Information Fig. S2)—suggest active defense response(s) to drought that are not controlled solely by source-driven processes or increased temperature. This is consistent with studies that have shown genes involved in biotic stress defenses to be upregulated in response to drought stress with concurrent downregulation of genes involved in cell division and growth (Dubos and Plomion 2003; Behringer et al. 2015; Moran et al. 2017). The severity and duration of stress required to exceed the physiological threshold(s) and initiating such responses remains relatively unknown for many species. Yet our data suggest that some stress level was reached and initiated a shift in the demand for monoterpenes. In fact, another study at our site showed that for two months prior to our first monoterpene sampling date, both *g*_s_ and net assimilation rates in the combined heat and drought stressed trees were significantly lower than ambient (Garcia-Forner et al. 2016). This is similar to patterns observed in *Pinus sylvestris* where monoterpenes only increased following periods of reduced *A*_max_ and *g*_s_ (Sancho-Knapik et al. 2017). Thus, while monoterpene synthesis may be decoupled from instantaneous *A*_max_, relatively short and intense periods of reduced net assimilation rates can lead to a reprioritization of carbon from NSCs towards biosynthetic pathways associated with chemical defense that is sustained for extended periods.

### Non-structural carbohydrates and total monoterpenes

With a significant decrease in photosynthetic rate, conifers rely on NSCs as a carbon source (Hartmann et al. 2013; Sevanto et al. 2014) and can synthesize carbon-based monoterpenes at the cost of storage and other secondary metabolites (Huang et al. 2019a). As hypothesized, increases in total monoterpene concentrations in woody tissues under water-limiting conditions were correlated with NSCs, namely fructose and glucose likely mobilized via starch hydrolysis (Fig. 5b and c). These observations indicate a link between chemical defenses and whole-tree carbon and water economics due to the spatiotemporal variation that exists in NSC storage and transport. These links, however, appear to be tissue specific and manifest themselves over different environmental conditions and time scales.

Specific plant tissues are known to possess different NSC allocation strategies that not only help cope with drought but can have important consequences for chemical defenses. Total monoterpenes in needles were not significantly correlated with total sugars, which explained some 23% of the variation in monoterpene concentrations (Table 2). This relatively poor relationship is likely due to the multifunctionality of sugars in leaf tissues where high quantities of soluble sugars are required to maintain normal cellular turgor (DeSchepper and Steppe 2011) while simultaneously supporting respiration and other critical processes. This weak correlation also suggests that other unmeasured carbon sources are supporting elevated monoterpene levels in the leaves under drought conditions (Fig. 2a). In contrast to leaf tissues in our study, total monoterpenes in the woody tissues were related to the previous month’s starch and glucose + fructose content (Fig. 5b and c), which explained more than 40% and 60% of the variation, respectively. These data suggest that drought-induced monoterpene synthesis in woody tissues is in part supported by starch hydrolysis as a source of soluble sugar substrates among other intermediate and stored carbon sources, the dynamics of which have yet to be identified. The delayed availability of glucose from starch (via starch synthesis, followed by hydrolysis and subsequent sugar transport to sites of monoterpene synthesis) may also explain the lag observed for concentration changes in these tissues (Table 2 and Fig. 3b).

While the NSCs measured did not explain more than 18% of the variation in drought-induced changes in individual monoterpene concentrations (Table 4 and Supporting Information Table S4), this does not preclude that other NSCs contribute to their synthesis. In addition, trade-offs between defenses and carbohydrate reserves may only manifest under more profound carbon limitations (Zust and Agrawal 2017) that were not reached in the present field-based study (McDowell et al. 2019). Nonetheless, these tissue-, organ- and compound-specific relationships with NSCs suggest synthesis controls beyond passive carbon source-sink dynamics, likely involving drought-induced phytohormonal signaling and the active gene regulation of specific monoterpene synthases (Radwan et al. 2017).

### Compositional responses of monoterpenes to heat and drought stress

Drought affects secondary compound synthesis via changes in source-sink carbon dynamics and also by altering the activities of key enzymes responsible for producing individual compounds like monoterpene synthases (Keeling and Bohlmann 2006). We observed compound- and tissue-specific changes in individual monoterpene concentrations and overall composition in response to the treatments (Table 3, Fig. 6). Notably, the differences in monoterpene profiles between trees exposed to the treatments and controls were greater in the woody tissue than in needles (Fig. 6). While it is common for chemical composition to differ between organs (Sjödin et al. 1996, 2000), the ways in which drought, heat, and their combination affect chemical diversity is less understood. It is possible that drought and heat affect monoterpene composition more in woody tissues as opposed to young needles due to either constraint on carbon transport (Sevanto 2014) and/or different biosynthetic processes in the tissue types (Manninen et al. 2002). Carbon is not allocated to individual compounds equally and is likely related to shifts in synthase enzyme activity that are differentially induced by heat and drought stress (Radwan et al. 2017). Because monoterpenes originate from the same precursor molecule, geranyl diphosphate, it is likely that these stress-induced shifts in individual concentrations are due to synthase activation, a topic that requires additional research. Identifying molecular mechanisms responsible for heat- and drought-induced shifts in monoterpene diversity will require a combination of transcriptomics, metabolomics, and stable isotope approaches, and our findings provide key insights that can be used to develop testable hypotheses to explain these targeted allocation patterns.

It is widely accepted that conifers become more susceptible to biotic attacks during drought due to decreased levels of defense compounds (Mattson and Haack 1987; Dobbertin et al. 2007; McDowell et al. 2011; Gaylord et al. 2013; Netherer et al. 2015). We identified decreased concentrations of some compounds despite overall increases in major constituents of the monoterpene profile. Drought-induced shifts in individual compounds may result from cumulative past evolutionary pressures, including drought-induced bark beetle attacks, especially given the unique changes to monoterpene profiles in woody tissue (Fig. 6). For example, drought and combined heat and drought stress increased levels of woody tissue (−)-α-pinene (Fig. 8a), a precursor molecule used by *I. confusus* to produce *cis*-verbenol, a minor constituent of the bark beetle’s pheromone blend that also consists of monoterpenoid alcohols (ipsenol and ipsdienol) synthesized de novo from the beetles themselves (Tittiger and Blomquist 2016; Fisher et al. 2021). In addition, our combined heat and drought treatments decreased levels of other woody tissue compounds, most notably (−)-δ-3-carene (Fig. 8d). High levels of δ-3-carene are characteristic of more resistant trees (Boone et al. 2011; Erbilgin et al. 2017) due to toxicity to bark beetles (Raffa and Berryman 1987; Raffa et al. 2005) and negative effects on the growth of symbiotic fungi (Raffa and Berryman 1983). While the effects of δ-3-carene on *I. confusus* are yet to be determined, the impact of this particular compound on both aggressive and non-aggressive bark beetle-fungi complexes suggests that its toxicity may be relatively common among taxa. Given the benefit of (−)-α-pinene as an aggregation pheromone precursor for *I. confusus* and the general toxicity of δ-3-carene to the bark beetle-fungal complex, higher (−)-α-pinene and lower δ-3-carene levels under combined heat and drought conditions may make these trees *more favorable* for bark beetle attack, rather than well-defended, which one might conclude if only studying monoterpene concentrations in total. Whether these shifts are enough to promote attack by *Ips confusus* (piñon engraver beetle) requires field studies investigating these mechanisms under more severe drought stress as well as bioassays that can provide a clearer understanding of how these altered profiles impact bark beetle choice and success.

## Conclusions

Two of the more important abiotic stressors affecting tree function are heat and drought, yet adequate tests of defense responses in mature trees to these stressors are rare and often fail to consider their interactive effects. Our experimental design allowed us to observe heat and drought-induced changes in monoterpenes in relation to growth, C assimilation, and NSCs over a range of water potentials that provided an opportunity to evaluate mechanistic underpinnings in the context of theory (GDBH) and potential impacts on destructive biotic agents. Our results point towards drought-induced changes in monoterpenes where cumulative climatic effects activate a reprioritization of source and sink strengths and thus allocation towards defense. While some aspects of our findings are consistent with the predictions of the GDBH, our results challenge source-based processes and suggest sophisticated signaling mechanisms are at play that actively reallocate NSCs to specific defense compounds after critical physiological thresholds induced by drought are surpassed. We found the interactive effects of heat and drought on monoterpenes to be frequently synergistic and sustained, although some compounds were insensitive or decreased in response to either stress across the levels we observed. This is critical to note as particular compounds play important roles in defense against biotic attacks. As such, it is imperative that the dynamics identified here continue to be studied to identify when key defensive compounds decline as drought becomes more severe, and how this occurs through NSC availability via gene regulation. It is clear that drought and heat stress change plant metabolism at levels ranging from enzymes to whole organisms. Coupling advanced metabolomics techniques to field-based experimental research may further illuminate the changes that plants undergo to defend against pest and pathogen attack while under physiological stress.

## Supplementary Information

Below is the link to the electronic supplementary material.

Supplementary file1 (DOCX 11783 KB)

## Data Availability

The data that support the findings of this study are openly available in Figshare.
