# Supplementary material for: Hotter droughts alter resource allocation to chemical defenses in piñon pine
Source: Oecologia. 2021 Oct 17;197(4):921–38. doi: 10.1007/s00442-021-05058-8 (PMC8591002; doi:10.1007/s00442-021-05058-8)
Supplement: Supplementary file 1 — Supplementary file1 (DOCX 11783 KB) [file 442_2021_5058_MOESM1_ESM.docx]

## Supporting Information

Article title: Hotter droughts alter resource allocation to chemical defenses in piñon pine

Authors: Amy M. Trowbridge, Henry D. Adams, Adam Collins, Turin Dickman, Charlotte Grossiord, Megan Hofland, Shealyn Malone, David K. Weaver, Sanna Sevanto, Paul C. Stoy, Nate G. McDowell

The following Supporting Information is available for this article:

**Table S1** Percentages [Mean (SEM)] of individual monoterpenes in *Pinus edulis* needle tissue for each of the four treatment groups over nine sampling periods (2012-2016).

Compound Ambient Heat Drought Heat+Drought

(-)-α-Pinene 20.71(2.50) 17.71(2.30) 19.89(2.50) 18.32(2.77)

(+)-α-pinene 25.56(4.88) 22.89(4.49) 29.19(4.87) 32.87(5.41)

tricyclene 0.24(0.05) 0.32(0.05) 0.21(0.05) 0.25(0.06)

(-)-camphene 0.05(0.02) 0.06(0.01) 0.03(0.02) 0.03(0.02)

(+)-camphene 0.007(0.001) 0.007(0.001) 0.006(0.001) 0.006(0.001)

β-myrcene 5.70(0.99) 7.24(0.92) 4.67(0.99) 5.07(1.11)

(-)-β-pinene 15.91(1.00) 10.65(0.95) 17.56(1.00) 12.57(1.08)

δ-3-carene 0.66(0.09) 0.55(0.09) 0.35(0.09) 0.37(1.00)

S-(-)-limonene 0.73(0.11) 1.01(0.10) 0.61(0.11) 0.73(0.12)

R-(+)-limonene 0.42(0.06) 0.40(0.06) 0.52(0.06) 0.48(0.07)

β-phellandrene 21.61(5.64) 29.44(5.17) 21.40(5.65) 25.24(6.30)

γ-terpinene 0.08(0.02) 0.13(0.02) 0.05(0.02) 0.10(0.02)

terpinolene 0.27(0.04) 0.26(0.04) 0.24(0.04) 0.26(0.05)

bornyl acetate 8.08(2.52) 9.01(2.31) 5.25(2.52) 3.68(2.80)

**Table S2** Percentages [Mean(SEM)] of individual monoterpenes in *Pinus edulis* woody tissue for each of the four treatment groups over nine sampling periods (2012-2016).

Compound Ambient Heat Drought Heat+Drought

(-)-α-Pinene 5.04(0.47) 5.94(0.45) 6.34(0.46) 5.76(0.50)

(+)-α-pinene 61.00(5.27) 75.26(4.84) 76.53(5.26) 79.37(5.87)

(-)-camphene 0.21(0.01) 0.27(0.01) 0.26(0.01) 0.27(0.01)

(+)-camphene 0.39(0.03) 0.49(0.03) 0.50(0.03) 0.51(0.04)

β-myrcene 9.95(2.12) 6.22(1.97) 7.97(2.11) 7.13(2.34)

(+)-β-pinene 0.45(0.07) 0.47(0.07) 0.46(0.07) 0.44(0.08)

(-)-β-pinene 2.23(0.76) 1.49(0.71) 2.51(0.76) 2.02(0.84)

δ-3-carene 16.04(3.39) 5.91(3.10) 1.76(3.38) 1.30(3.78)

S-(-)-limonene 0.19(0.02) 0.15(0.01) 0.14(0.01) 0.14(0.02)

R-(+)-limonene 0.31(0.02) 0.37(0.02) 0.38(0.02) 0.40(0.02)

β-ocimene 0.67(0.13) 0.98(0.13) 0.65(0.13) 0.58(0.15)

β-phellandrene 0.80(0.20) 0.61(0.18) 0.74(0.20) 0.64(0.22)

γ-terpinene 0.21(0.04) 0.04(0.04) 0.09(0.04) 0.06(0.05)

terpinolene 1.76(0.26) 0.62(0.24) 0.65(0.26) 0.41(0.29)

bornyl acetate 0.82(0.06) 0.90(0.06) 0.97(0.06) 0.96(0.06)

**Table S3**

F-values, degrees of freedom, and P-values of PERMANOVA analyses of the effects of treatment, time, and their interaction on monoterpene composition of needle and woody tissue of *Pinus edulis* averaged over nine sampling periods (2012-2016).

| Tissue | F-value | DF | *P*-value | R^2^ |
| --- | --- | --- | --- | --- |
| Needle |  |  |  |  |
| Treatment | 10.93 | 3,140 | <0.001** | 0.20 |
| Time | 1.33 | 8,140 | <0.001** | 0.07 |
| Treatment*Time | 0.55 | 24,140 | 0.30 | 0.08 |
| Woody |  |  |  |  |
| Treatment | 26.08 | 3,142 | <0.001** | 0.33 |
| Time | 2.86 | 8.142 | <0.001** | 0.10 |
| Treatment*Time | 1.19 | 24,142 | 0.012* | 0.12 |

**Table S4** Repeated measures correlation analyses of individual monoterpene compound concentrations with non-structural carbohydrates (starch, sucrose, and glucose + fructose) from needle tissue of *Pinus edulis* across all sampling dates throughout 2012-2016. Repeated measures r_m_ correlation coefficient(*P*-values) is displayed, and significant relationships are indicated in bold after applying the Bonferroni correction for multiple comparisons (α <0.0012).

| Compound | Starch | Sucrose | Glucose + Fructose |
| --- | --- | --- | --- |
| (-)-α-pinene | −0.14(0.16) | −0.03(0.78) | −0.008(0.44) |
| (+)-α-pinene | −0.16 (0.10) | −0.12(0.24) | −0.04(0.71) |
| tricyclene | −0.21(0.04) | −0.36(0.0002) | 0.28(0.004) |
| (-)-camphene | −0.27(0.007) | −0.31(0.001) | 0.27(0.007) |
| (+)-camphene | −0.31(0.002) | −0.31(0.001) | 0.16(0.10) |
| β-myrcene | −0.01(0.89) | 0.20(0.05) | −0.04(0.72) |
| β-pinene | −0.16(0.12) | −0.07(0.51) | −0.08(0.41) |
| δ-3-carene | −0.10(0.35) | 0.03(0.77) | −0.02(0.82) |
| (-)-limonene | −0.21(0.04) | −0.25(0.01) | 0.23(0.02) |
| (+)-limonene | −0.20(0.05) | −0.23(0.02) | 0.04(0.68) |
| β-phellandrene | −0.25(0.013) | −0.32(0.001) | 0.22(0.03) |
| γ-terpinene | −0.12(0.24) | −0.35(0.0002) | **0.42(9×10^−6^)** |
| terpinolene | −0.02(0.81) | 0.09(0.37) | 0.17(0.08) |
| bornyl acetate | −0.20(0.06) | −0.19(0.05) | 0.22(0.02) |

**Fig. S1** Fresh weight (FW): Dry weight (DW) coefficients for *Pinus edulis* needles (a) and woody tissue (b) across the four treatment types (A: ambient, H: heat, D: drought, and H + D: heat and drought) averaged over nine sampling periods from 2012 to 2016. Means ± SEM are shown and significant differences between treatments is expressed using differing lower-case letters (α <0.05).

**
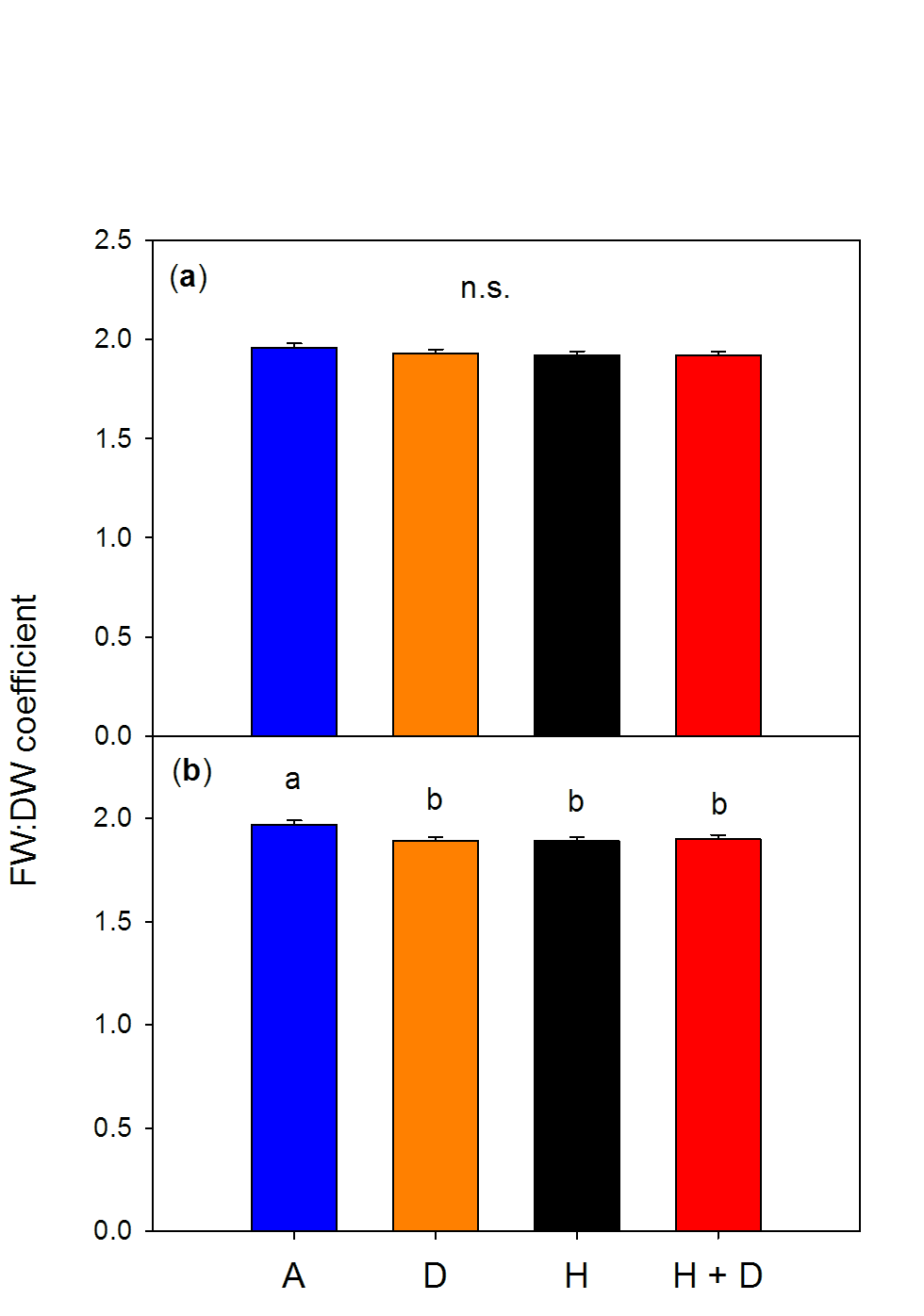
**

**Fig. S2** Time series data representing mean (± SEM) annual primary growth (mm) of *Pinus edulis* shoots for trees exposed to ambient (blue circles), heat (orange circles), drought (black circles), and heat + drought (red circles) from 2010 - 2016. Dashed line represents the establishment of the treatments and different colored asterisks represent a significant difference from ambient for that particular treatment.


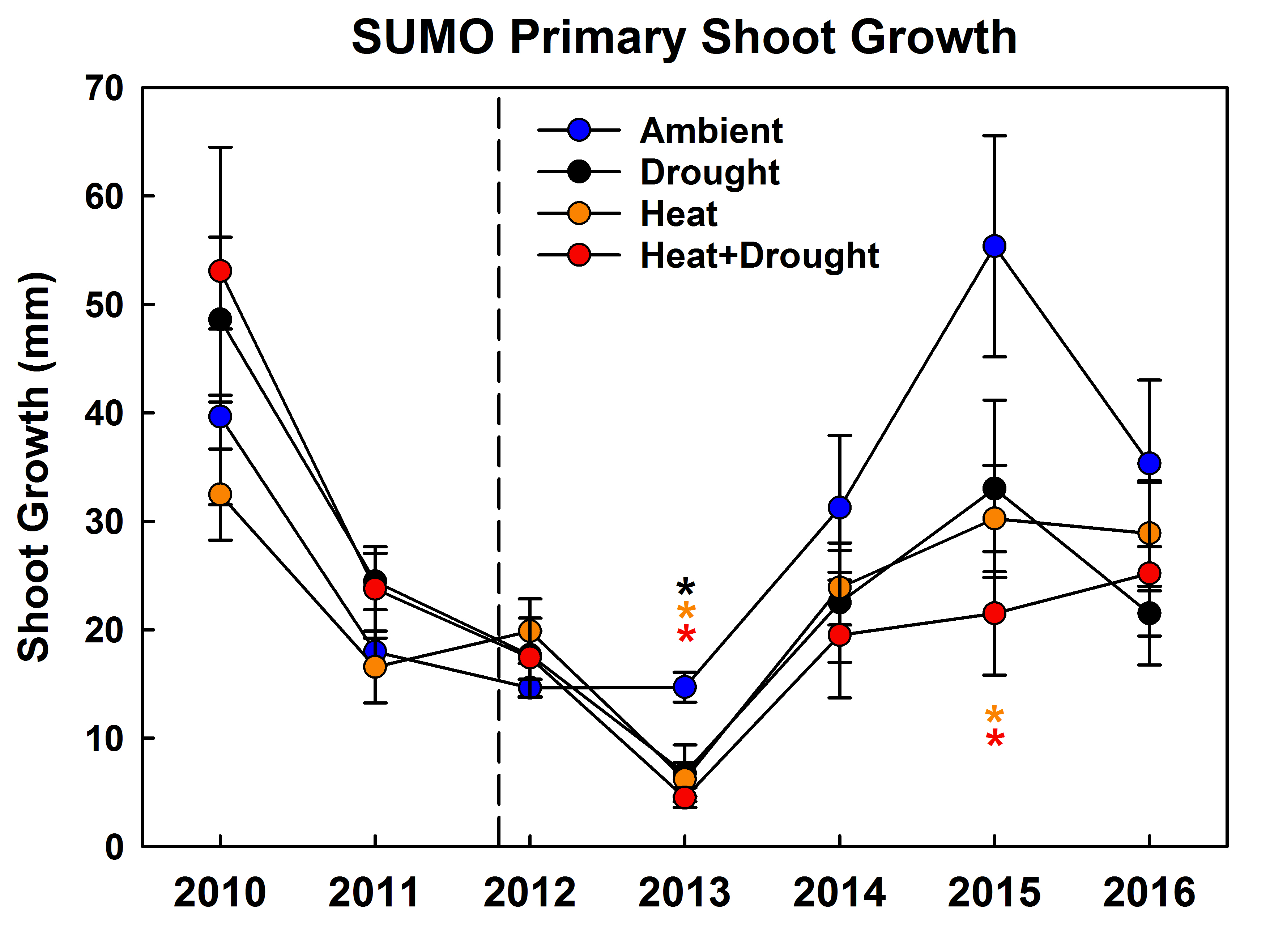


**Fig. S3** Total monoterpene compound concentrations (mg g DW^-1^) in *Pinus edulis* needles (a) and woody tissue (b) across the four treatment types (A: ambient, H: heat, D: drought, and H + D: heat and drought) averaged over nine sampling periods from 2012 to 2016. Means ± SEM are shown and significant differences between treatments is expressed using differing lower-case letters (α <0.05).

**
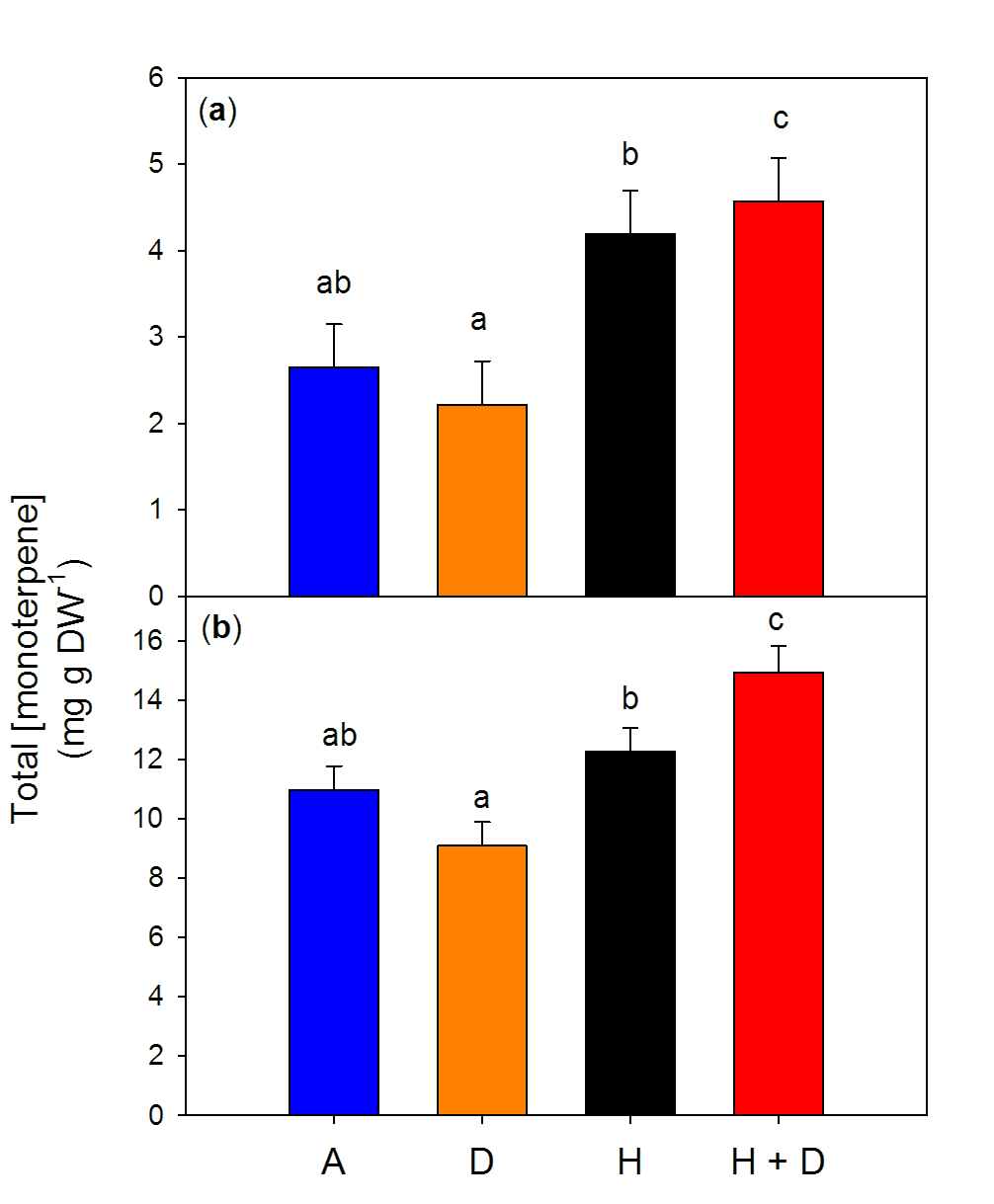
**

**Fig. S4** Pair-wise comparison of dispersion of monoterpene composition of *Pinus edulis* in needles (a) and woody tissue (b) for four treatments types (A: ambient, H: heat, D: drought, and H + D: heat and drought) averaged over nine sample periods (2012-2016). Mean differences are surrounded by bias-corrected 95% confidence intervals; significant differences in dispersion are indicated by 95% confidence intervals that do not overlap zero.

**Methods S1** Sample processing and chemical analysis of monoterpenes

For monoterpene extraction, needles were ground in liquid nitrogen using a chilled mortar and pestle to minimize monoterpene loss. Frozen needle powder was then weighed into 2-dram glass vials, exact weights (between 0.4 and 0.6 g) were recorded, and 4 mL of GC-grade n-hexane (Fisher Scientific) containing 0.1 µL mL^-1^ (+)-fenchone (Sigma-Aldrich) as an internal standard was added to each vial. Frozen stems were processed in the same manner as the needles, but rather than grinding them into a powder, woody tissues were cut into 2-4 mm pieces to expose resin ducts for the extraction process. Vials were immediately closed with PTFE lined caps, mixed with a vortexer, and allowed to soak for 7 days at ambient temperature. After the soaking period, 100 µL the solution from each monoterpene sample was injected into micro-inserts in small mouth clear GC vials and capped with PTFE liners (Alltech Associates, Deerfield, IL). Chemical analysis was then performed using using gas chromatography-mass spectrometry (GC-MS). Remaining samples were then stored in the freezer for future use. One µL of each sample was injected onto an Agilent Technologies 6890 gas chromatograph-5975 mass spectrometer (GC-MS) fitted with a Cyclodex-B chrial column (30 m x 250 µm x 0.25 µm; J&W Scientific). Helium was used as the carrier gas at a flow rate of 1.0 mL min^-1^ with a split flow ratio of 25:1. Injector temperature was set at 230 °C. The oven profile consisted of an initial temperature of 60 °C followed by a ramp of 5 °C min^-1^ to 200 °C, then a second ramp of 25 °C min^-1^ to 230 °C. Monoterpene enantiomers were identified by comparing retention times of known standards and mass spectra using the NIST library and MSD Chemstation software. Concentrations for each compound were calculated using four-point calibration curves with injections of known amounts of pure standards and the internal standard, fenchone. All standards were purchased from Sigma Aldrich (Saint Louis, MO) with the exception of β-phellandrene (Glidco Organics, Jacksonville, FL). Subsamples of needles and woody tissue from each sample collected from each tree were used to calculate fresh weight to dry weight ratios (FW:DW) to account for potential variation in concentrations due to leaf water content. Comparative statistics showed that dry weight conversion did not significantly change the results so all data are presented on per fresh mass basis.
